# Alveolar Macrophages Isolated Directly From Human Cytomegalovirus (HCMV)–Seropositive Individuals Are Sites of HCMV Reactivation In Vivo

**DOI:** 10.1093/infdis/jiu837

**Published:** 2014-12-30

**Authors:** Emma Poole, Jatinder K. Juss, Benjamin Krishna, Jurgen Herre, Edwin R. Chilvers, John Sinclair

**Affiliations:** Department of Medicine,University of Cambridge,United Kingdom

**Keywords:** human cytomegalovirus, macrophages, reactivation

## Abstract

Human cytomegalovirus (HCMV) causes significant morbidity in the immunocompromised host. Following primary infection, the virus establishes latent infection in progenitor cells of the myeloid lineage. These cells exhibit limited viral gene transcription and no evidence of *de novo* virion production. It is well recognized that differentiation of latently infected myeloid progenitor cells to dendritic or macrophage-like cells permits viral reactivation in vitro. This has been used to support the concept that viral reactivation in HCMV carriers routinely occurs from such terminally differentiated myeloid cells in vivo. However, to date this has not been shown for in vivo–differentiated macrophages. This study is the first to demonstrate that alveolar macrophages from HCMV carriers express immediate early lytic genes and produce infectious virus. This supports the view, until now based on in vitro data, that terminally differentiated myeloid cells in vivo are sites of HCMV reactivation and potential centers of viral dissemination in latently infected individuals with no evidence of virus disease or dissemination.

Human cytomegalovirus (HCMV) has the largest genome among human herpesvirus, and, like all herpesviruses, it has 2 different life cycles. The lytic life cycle occurs in terminally differentiated cells such as fibroblasts, dendritic cells (DCs), and endothelial cells and commences with immediate early (IE) gene expression, followed by early gene expression and late gene expression. This culminates in viral production over a 72-hour period, as observed in primary fibroblasts in culture [1]. Primary HCMV infection of immunocompetent individuals rarely results in serious disease. Despite a robust immune response, HCMV is not eradicated in these individuals, as the virus enters a latent phase of infection [2–4]. Cells of the myeloid lineage, such as CD34^+^ progenitor cells and their CD14^+^ monocyte derivatives, are well-characterized sites of HCMV latency in healthy seropositive carriers. Differentiation of these naturally latently infected myeloid cells ex vivo into terminally differentiated DCs leads to reactivation of the viral lytic transcription program and virion production [2–6]. Similarly, this differentiation-dependent reactivation of latent virus can also be fully recapitulated in a number of experimental models of HCMV latency in CD34^+^ or CD14^+^ cells infected in vitro [7–11]. Collectively, these observations of the lytic and latent life cycles of HCMV in the myeloid lineage underscore the close link between myeloid differentiation and virus reactivation from latency and predict that reactivation of the viral lytic transcription program should occur routinely in terminally differentiated myeloid cells in vivo. Consistent with this, recent work has shown that immature DCs taken directly from the blood of HCMV-seropositive individuals are positive for viral IE gene expression and reactivate full virus production under additional inflammatory stimuli ex vivo [12].

In addition to their differentiation into DCs, the differentiation of monocytes into monocyte-derived-macrophage (MDM) cell lines ex vivo produces cells that are permissive to HCMV infection [13–15]. Additionally, reactivation of lytic gene expression from latently infected myeloid cells differentiated ex vivo into MDMs has also been demonstrated [16, 17], but whether macrophages differentiated in vivo (eg, alveolar macrophages) are also sites of virus reactivation has never previously been addressed.

HCMV pneumonitis with virus detectable in bronchoalveolar lavage (BAL) can occur after both solid organ and bone marrow transplantations [18], and this has suggested that the lung may be a normal site of reactivation in healthy carriers in vivo, which, in the transplant recipient, cannot be limited by the normal host immune response due to recipient immunosuppression. We are the first to show that macrophages differentiated in vivo (ie, alveolar macrophages) are sites of reactivation of HCMV in virus carriers in the absence of HCMV disease or inflammation. Collectively, these data argue that the differentiation of myeloid precursor cells in vivo into alveolar macrophages promotes the reactivation of HCMV lytic gene expression in individuals who are not viremic for HCMV disease and helps confirm findings from studies performed on HCMV reactivation that used ex vivo generation of macrophages from myeloid precursors.

## METHODS AND METHODS

### Ethics Statement

All human studies were approved by the local research ethics committee and complied with the Declaration of Helsinki. Written informed consent to collect blood and to retain BAL fluid (BALF) was obtained from patients undergoing fiber-optic bronchoscopy (ethical approval UK08/H0306/17 and UK06/Q0108/281).

### Isolation and Purification of Alveolar Macrophages From BALF

BALF was obtained from routine bronchoscopies for noninflammatory/noninfectious disorders, with the BAL taken from radiologically normal lobes. The fiber-optic bronchoscopy was wedged into the subsegmental bronchus in an area contralateral to the side of any radiological change observed on the chest radiograph or computed tomogram. A total of 150 mL of sterile isotonic saline was instilled in 50-mL aliquots with gentle suctioning after each aliquot. BALF recovery averaged 90 mL (60%), with a range of 50 to 120 mL. The retrieved BALF was immediately placed on ice and processed within 15 minutes by filtering through sterile gauze to remove mucus. The BALF was centrifuged at 300*g* at 4°C for 7 minutes, and the cell pellet was washed twice in 40 mL of phosphate-buffered saline with calcium and magnesium and resuspended at 5 × 10^5^ cells/mL in X-vivo-15 (Lonza) supplemented with 1:100 Gibco Antibiotic-Antimycotic (containing penicillin 10 000 U/mL, streptomycin 10 000 U/mL, and 25 μg/mL amphotericin B from Life Technologies15 240 062). The differential leukocyte count of the BALF was determined by light microscopy of cytospins stained with modified Wright stain (WS16; Sigma-Aldrich). The cells isolated from the BALF were cytocentrifuged for 3 minutes at 300*g* onto Polysine microscope slides (VWR International), using a Shandon Cytospin 2 (Shandon, United Kingdom). Alveolar macrophages were identified by their large dark nuclei and abundant pale, granular cytoplasm containing numerous vacuoles. Alveolar macrophages composed 95% ± 2% of the BALF differential leukocyte count. All patients were negative for C-reactive protein, confirming lack of inflammation.

### HCMV Immunoglobulin G (IgG) Serotyping of Donors

HCMV-specific IgG was assessed in patients’ serum specimens by the Capita Cytomegalovirus IgG enzyme-linked immunosorbent assay (ELISA) kit (Trinity Biotech) according to the manufacturer's instructions.

### HCMV Immunoglobulin M (IgM) Serotyping of Donors

Donor serum specimens were tested using the Liaison CMV IgM II chemiluminescence immunoassay (DiaSorin) for the semiquantitative determination of specific IgM antibodies to HCMV in human serum or plasma samples, according to the manufacturer's protocol.

### Polymerase Chain Reaction (PCR) Analysis to Detect DNA Viremia

Nucleic acid extractions from serum specimens were performed on the automated BioRobot MDx extractor, using the One-for-All kit. CMV RNA was quantified relative to the GAPDH housekeeping gene, using the primers 5′-TGCCGGGTGGCGAGTACCCTGT-3′ (forward) and 5′-CAGTTCCGAGAGCACCGAGACG-3′ (reverse), with the probe 5′-[FAM]ATACAGCGTCACGCTAG[MGB]-3′. The following PCR parameters were used: for the reverse transcription (RT) step, 50°C for 3 minutes, followed by denaturation at 95°C for 3 minutes; and for the PCR step, 50 cycles at 95°C for 8 seconds and 58°C for 60 seconds. The samples were analyzed on the Rotor-Gene cycler.

### RT–Quantitative PCR (qPCR) Analysis of IE Expression in Alveolar Macrophages

Purified alveolar macrophages were lysed directly in Trizol (Invitrogen), and RNA was isolated using a micro RNA purification kit (Qiagen). RNA was quantified relative to the GAPDH housekeeping gene, using the following primers and probes: for IE, primers CAAGAACTCAGCCTTCCCTAAGAC and TGAGGCAAGTTCTCGAATGC, with the probe [FAM]CCAATGGCTGCAGTCAGGCCATG[TAM]; for GAPDH, primers GGAAGCTTGTCATCAATG and CCCCACTTGATTTTGGAG, with the probe [JOE]ATCACCATCTTCCAGGAGCGAG[BHQ1]; and for UL138, primers 5′-CGCTGTTTCTCTGGTTAG and 5′-CAGACGATACCGTTTCTC, with the probe CCGACGACGAAGACGATGAAC[Cy5]. The following PCR parameters were used: for the RT step, 50°C for 20 minutes, followed by heat-based inactivation at 95°C for 5 minutes; and for the PCR step, 50 cycles at 95°C for 15 seconds and 60°C for 45 seconds. The samples were analyzed on an ABI Real-Time 7500 machine.

### ELISA of Tumor Necrosis Factor α (TNF-α) Levels

BALF fluid was tested for levels of TNF-α, using the RND Systems ELISA kit according to the manufacturer's protocol.

### Coculture of Alveolar Macrophages and Fibroblasts

Purified alveolar macrophages (5 × 10^5^ macrophages/well) were cultured in a 24-well plate overnight before overlaying with fibroblasts (1 × 10^5^ macrophages/well). After 1 week, supernatants were transferred onto fresh human foreskin fibroblasts to quantify virus levels in the supernatant. After 3 weeks, the cells were fixed in 70% ethanol and stained for IE by immunofluorescence as previously described [19].

## RESULTS

### Alveolar Macrophages Are Permissive to HCMV Lytic Infection

**Figure 1.**
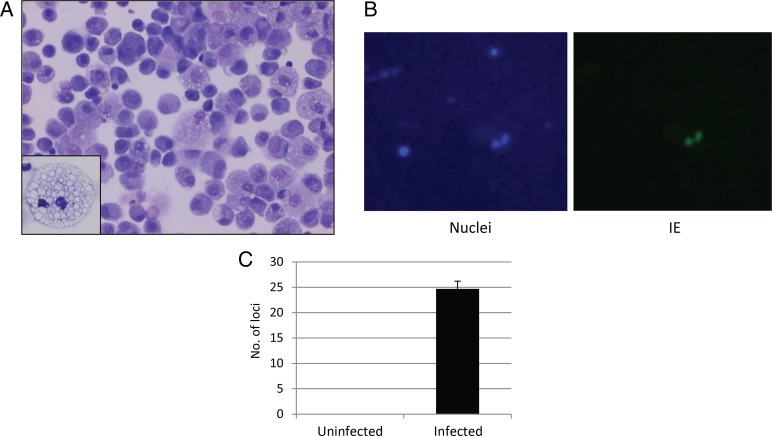
Alveolar macrophages ex vivo from individuals are permissive to lytic human cytomegalovirus (HCMV) infection. *A*, Representative photomicrograph (original magnification ×40) of a typical cytospin preparation of freshly isolated human alveolar macrophages stained with modified Wright stain. Alveolar macrophages were identified by their large dark nuclei and abundant pale, granular cytoplasm containing numerous vacuoles. Alveolar macrophages typically constitute >95% of the differential leukocyte count in bronchoalveolar lavage fluid. *B*, Isolated macrophages were adhered to plastic and then infected with the TB40E-IE-YFP strain of HCMV at a multiplicity of infection of 5. After 24 hours, some cells were counterstained with Hoechst stain to detect nuclei. *C*, Alternatively, following infection of the macrophages with TB40E-IE-YFP virus, cells were washed and left for 1 week, and the supernatant was collected and transferred onto fresh fibroblasts. Abbreviation: IE, immediate early.

A strong link between the permissiveness of cells to in vitro infection with HCMV and their ability to reactivate latent virus has previously been established [2, 3]. We therefore assessed whether in vivo–differentiated alveolar macrophages could support full HCMV lytic infection. Although it was demonstrated that alveolar macrophages matured in vitro were permissive to HCMV infection [13, 15, 16], in vivo–differentiated alveolar macrophages have not, to our knowledge, been tested. Alveolar macrophages were harvested to 95% purity, as assessed by differential cell counts, from the BALF of 10 donors (Figure 1*A*). After adherence to plastic, cells were infected at a multiplicity of infection of 5 with TB40E-IE-YFP virus, which expresses YFP-tagged IE86 and allows live detection of virus infection. Figure 1*B* shows that, 24 hours after infection with virus, IE protein was detectable in the nuclei of infected macrophages. Furthermore, supernatants taken from the macrophages 1 week after the start of HCMV infection showed virus transfer onto fresh fibroblasts (Figure 1*C*). Therefore, in vivo–differentiated alveolar macrophages are fully permissive to HCMV infection ex vivo.

### Alveolar Macrophages From Seropositive Individuals Are Sites of Viral IE Gene Expression

**Figure 2.**
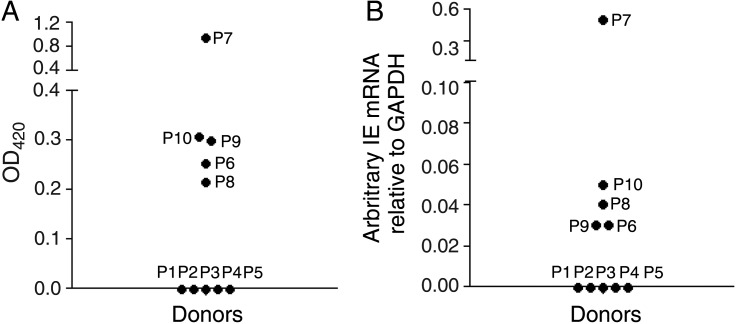
Alveolar macrophages from seropositive individuals express immediate early gene (IE) messenger RNA (mRNA). *A*, Serum specimens from 10 donor patients (P) were tested for the presence of IE immunoglobulin G (IgG) by enzyme-linked immunosorbent assay (ELISA). *B*, Alveolar macrophages were isolated directly ex vivo from the same patients and harvested for reverse-transcription quantitative polymerase chain reaction (PCR) analysis for the presence of human cytomegalovirus (HCMV) IE mRNA. A blood specimen from each patient was also assessed by PCR for the presence of HCMV DNA, to determine whether viremia was present, and for the presence of IgM. These data and the data from panels *A* and *B* are summarized in Table 1.

In vitro infection of in vivo–differentiated macrophages clearly demonstrated that these cells were permissive to HCMV infection, suggesting that these differentiated macrophages may also be sites of reactivation in vivo. To test whether in vivo–differentiated macrophages express viral IE genes, serum specimens and alveolar macrophages were isolated from 10 donors for analysis. The serostatus (ie, the presence of HCMV-specific IgG) of each donor was assessed using ELISA. Figure 2*A* shows that 5 of 10 donors were seropositive.

Next, the expression of viral IE genes in alveolar macrophages from seropositive carriers was assessed using sensitive RT-qPCR. Figure 2*B* shows that all alveolar macrophages from seropositive donors were also positive for viral IE gene expression and that, as predicted, only seropositive donors showed viral gene expression. To confirm that this ability to detect viral IE RNAs in alveolar macrophages was not due to any ongoing HCMV viremia in the donor, all donors were tested directly for HCMV viremia, using 2 independent assays. HCMV-specific IgM levels in serum specimens were assessed. As hypothesized, HCMV-seronegative donors were also negative for HCMV-specific IgG (Table 1), and, with the exception of one donor (donor 7), none of the HCMV-positive donors were positive for HCMV-specific IgM or viral genome in blood. In contrast, donor 7, who presented with elevated levels of IgM antibodies to HCMV also had a blood specimen with a positive PCR result, arguing that this donor was likely viremic.

**Table 1.** Alveolar Macrophages From Seropositive Individuals Express Immediate Early Gene (IE) Messenger RNA

Patient	IE RNA	IgG	IgM	DNA, by PCR
1	Not detected	Not detected	Not detected	Not detected
2	Not detected	Not detected	ND	Not detected
3	Not detected	Not detected	Not detected	Not detected
4	Not detected	Not detected	Not detected	Not detected
5	Not detected	Not detected	Not detected	Not detected
6	Detected	Detected	ND	Not detected
7	Detected	Detected	Detected	9.79 × 10^4^ copies/mL
8	Detected	Detected	Not detected	Not detected
9	Detected	Detected	Not detected	Not detected
10	Detected	Detected	Not detected	Not detected

Abbreviations: IgG, immunoglobulin G; IgM, immunoglobulin M; ND, not done; PCR, polymerase chain reaction.

Collectively, these results provide clear evidence that in vivo–differentiated macrophages obtained from seropositive donors who show no signs of HCMV disease and are not viremic are routinely positive for viral IE expression, arguing that these are sites of HCMV IE gene reactivation in vivo.

As it was crucial to determine the inflammatory status of these individuals, further tests were performed to detect markers of inflammation both systemically (by the presence of C-reactive protein), as well as in the local microenvironment (by the presence of TNF-α). Table 2 shows that the tested individuals were not positive for C-reactive protein and that the BALF did not contain detectable levels of TNF-α when assessed by ELISA. These data suggest that any virus present in the alveolar macrophages in these patients was not due to a nonspecific inflammatory induction but was rather a naturally occurring reactivation event.

**Table 2.** Results of Tests for Detection of the Inflammation Markers Tumor Necrosis Factor α (TNF-α) and C-reactive Protein, by Human Cytomegalovirus Serostatus

Inflammation Marker	Positive Control	Seronegative	Seropositive
TNF-α, pg/mL	424 ± 21	Undetectable	Undetectable
C-reactive protein	NA	Negative	Negative

Abbreviation: NA, not applicable.

### In Vivo–Differentiated Alveolar Macrophages Are Sites of HCMV Virion Production

It is known that naturally latent monocytes can be differentiated to allow reactivation of viral IE gene expression but without subsequent viral gene expression or production of virions [2, 3, 7–11]. Similarly, a number of in vitro studies have demonstrated that several cell types can be abortively infected with HCMV; they support IE gene expression but not full virion production [2, 3]. Therefore, 2 donors, one seronegative and the other seropositive, were further analyzed to determine whether alveolar macrophages were natural sites of full virus reactivation and virion production.

**Figure 3.**
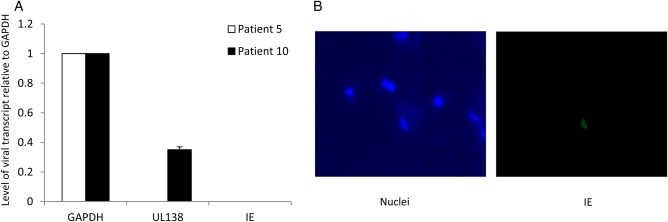
Monocytes ex vivo from a human cytomegalovirus (HCMV)-seropositive patient transcribe the HCMV latency gene UL138, and alveolar macrophages from the same donor express the HCMV lytic immediate early gene (IE) protein. Donors were tested for systemic inflammation (as revealed by the presence of C-reactive protein) and local bronchoalveolar fluid (BALF) inflammation (by means of enzyme-linked immunosorbent assay [ELISA] specific for tumor necrosis factor α [TNF-α]). A positive control for the ELISA was supernatant from monocyte-derived dendritic cells (DCs) shown in Table 2. *A*, Monocytes were isolated from venous blood and harvested for reverse-transcription quantitative polymerase chain reaction analysis. *B*, Isolated alveolar macrophages were cytospun and then fixed and stained for detection of the HCMV lytic antigen, IE. Cells were counterstained with Hoechst stain to detect nuclei.

We and others have previously shown that latent infection in healthy seropositive virus carriers can be assessed by the analysis of peripheral monocytes for HCMV lytic gene expression [2]. To ensure that the seropositive donor was latently infected, CD14^+^ monocytes were isolated from blood and analyzed for the standard hallmarks of latency: the presence of latent transcripts and the absence of lytic gene transcription. Figure 3*A* shows that CD14^+^ cells isolated from venous blood of the seropositive donor expressed the latency-associated transcript UL138 in the absence of expression of the lytic IE gene. We did not observe viral gene expression in monocytes from the seronegative donor. Alveolar macrophages from both donors were stained for detection of IE; IE expression was detectable in a few cells from the seropositive donor (Figure 3*B*) but in no cells from the seronegative donor (data not shown).

**Figure 4.**
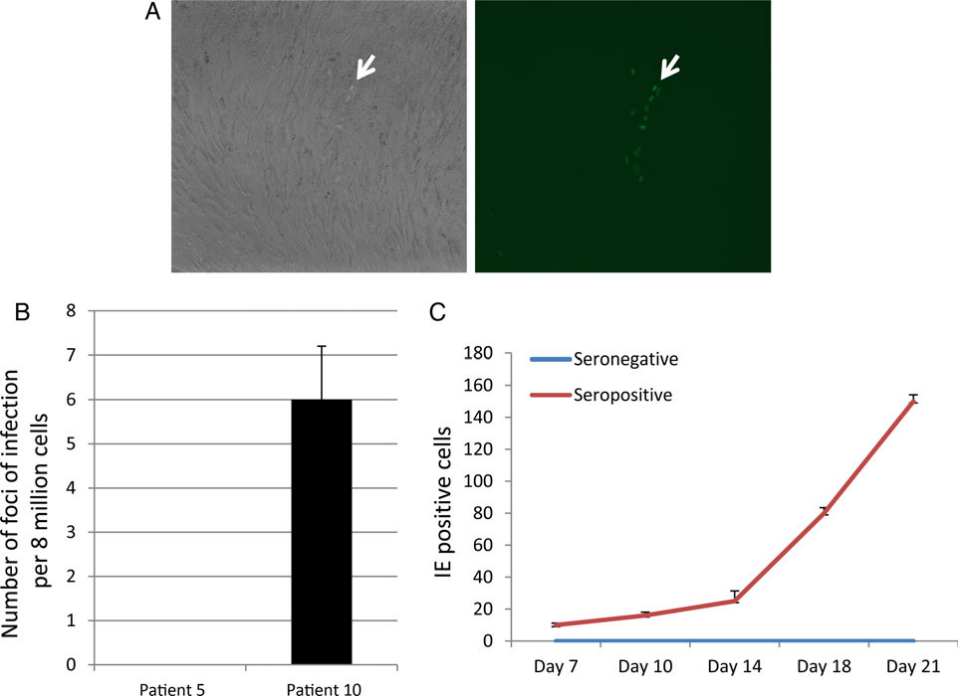
Alveolar macrophages isolated from human cytomegalovirus (HCMV)-seropositive donors support HCMV production and viral spread. *A*, Alveolar macrophages were cocultured with indicator fibroblasts and stained for immediate early gene (IE) by immune fluorescence. Light microscopy (left) and fluorescence (right) image of fibroblasts are shown; the green nuclei indicate IE-positive cells (arrow). *B* and *C*, The number of IE-positive foci were quantitated (*B*), and supernatants collected after coculture were transferred onto fresh monolayers of fresh human foreskin fibroblasts, with the number of IE-positive cells quantitated 24 hours after transfer (*C*).

To determine whether alveolar macrophages are sites of reactivation of full virus production, they were cocultured on indicator fibroblasts. After 3 weeks of coculture, the fibroblasts were fixed and stained for detection of infection foci where IE was being expressed. Figure 4*A* shows that foci of infection were clearly identifiable as areas of swollen cells within the fibroblast monolayer. Figure 4*B* shows that less than one in a million alveolar macrophages from seropositive individuals are productive for viral spread in vitro. Finally, to demonstrate that virions were being released into the supernatant, the supernatants were collected and transferred onto fresh fibroblasts, and the numbers of IE-expressing cells were enumerated over time (Figure 4*C*).

Collectively, these are the first data to show that alveolar macrophages from donors who are seropositive for HCMV but not viremic are fully competent sites of HCMV reactivation.

## DISCUSSION

The extent to which HCMV reactivation occurs in seropositive healthy carriers is uncertain. One hypothesis is that viral reactivation occurs at the molecular level routinely in healthy carriers but that dissemination of virus from these reactivation events is prevented by immune competence.

It has been postulated that such molecular reactivation of latent virus in vivo, in healthy carriers, results from the differentiation of myeloid progenitor cells to macrophages and DCs [2, 3, 7–9, 11, 17]. However, this hypothesis is largely based on the ex vivo differentiation of myeloid progenitors. Previous work demonstrated that differentiated but immature myeloid DCs from healthy seropositive HCMV carriers are sites of reactivation of HCMV IE gene expression in vivo but that reactivation of infectious virus required additional inflammatory cytokine treatment ex vivo [12]. Our results provide direct evidence that in vivo terminally differentiated alveolar macrophages are sites of full HCMV reactivation in virus carriers who do not show signs of HCMV disease, viremia, or inflammation either systematically or in the macrophage microenvironment.

Consistent with the view that differentiated macrophages could represent a supportive environment for productive HCMV infection, monocyte-derived macrophages are fully permissive to HCMV infection [13, 15, 16]. These findings are consistent with previous data that demonstrated that macrophages are sites of virus infections in individuals with acute HCMV disease [20]. Indeed, it has been demonstrated that the lung is important for reactivation and disease progression during HCMV infection [21]. Whether the IE expression observed in alveolar macrophages in this study results from de novo HCMV infection produced from other undefined cells following a reactivation event or whether it results from the reactivation of HCMV in the alveolar macrophages directly remains to be established. However, none of the donors exhibited signs of viremia or inflammation, suggesting that de novo infection of the alveolar macrophages was unlikely. Furthermore, there is a body of evidence from natural and experimental latent systems that shows that differentiation of myeloid progenitors into macrophage-like cells or DCs is an established mechanism for reactivation [2, 3, 7–9, 11, 17]. Establishment of HCMV latency can cause partial differentiation of monocytes [22, 23], and, although analyzed in an experimental latency model by using monocytes grown in differentiation cytokines and hence likely already partially differentiated, Noriega et al [24] have recently shown that latent infection itself can drive differentiation to a macrophage-like phenotype, in itself, aiding reactivation of virus. Thus, it seems likely that the observations presented here represent in vivo reactivation events in the myeloid lineage culminating in virus release from macrophages.

Our observations that in vivo–differentiated macrophages support the reactivation of latent virus also provide strong support for the analysis of experimental latency models, which is underway in a number of laboratories, to understand the mechanisms essential for the maintenance of latent HCMV and its subsequent reactivation in the cells of the myeloid lineage and, thus, validate proposed clinical and therapeutic applications [25]. One caveat is that the myeloid lineage may not be the only site of HCMV latency and reactivation [26]. Therefore, similar studies of other cells, such as neural cells and their progenitors, may be similarly informative.
